# Supplementary material for: MetaRibo-Seq measures translation in microbiomes
Source: Nat Commun. 2020 Jun 29;11:3268. doi: 10.1038/s41467-020-17081-z (PMC7324362; doi:10.1038/s41467-020-17081-z)
Supplement: Supplementary file 10 — Supplementary Data 7 [file 41467_2020_17081_MOESM10_ESM.zip › File2/Confidence_High_Taxonomy/158100_out.krona.html]

Javascript must be enabled to view this page.

members
magnitude
magnitudeUnassigned
count
unassigned
taxon
rank

158100\_out

12

12
superkingdom
2

12
32066
phylum

12
class
203490

203491
order
12

12
family
203492

3
genus
848

SRS014691\_contig\_number\_14197SRS074976\_contig\_number\_18151SRS104045\_contig\_number\_3437
12

1

SRS1055047\_contig\_number\_contig-100\_28.26748
1852365
species


SRS014468\_contig\_number\_contig-100\_712.712SRS017324\_contig\_number\_contig-100\_13315.47870SRS077508\_contig\_number\_contig-100\_11452.93351SRS149330\_contig\_number\_277
860
species
4

species
851
4


SRS017757\_contig\_number\_contig-100\_13907.120474.120474SRS1055037\_contig\_number\_88
subspecies
76857
2


SRS893335\_contig\_number\_1171
76859
subspecies
1


SRS015062\_contig\_number\_contig-100\_554.554
155615
subspecies
1
